# Supplementary material for: Spatially resolved cell atlas of the mouse primary motor cortex by MERFISH
Source: Nature. 2021 Oct 6;598(7879):137–43. doi: 10.1038/s41586-021-03705-x (PMC8494645; doi:10.1038/s41586-021-03705-x)
Supplement: Supplementary file 1 — Reporting Summary [file 41586_2021_3705_MOESM1_ESM.pdf]

nature research

Corresponding author(s): Xiaowei Zhuang

Last updated by author(s): May 30, 2021

Reporting Summary

Nature Research wishes to improve the reproducibility of the work that we publish. This form provides structure for consistency and transparency in reporting. For further information on Nature Research policies, see Authors & Referees and the Editorial Policy Checklist.

Statistics

For all statistical analyses, confirm that the following items are present in the figure legend, table legend, main text, or Methods section.

n/a	Confirmed	
☐	☒	The exact sample size (*n*) for each experimental group/condition, given as a discrete number and unit of measurement
☐	☒	A statement on whether measurements were taken from distinct samples or whether the same sample was measured repeatedly
☒	☐	The statistical test(s) used AND whether they are one- or two-sided<br>*Only common tests should be described solely by name; describe more complex techniques in the Methods section.*
☒	☐	A description of all covariates tested
☒	☐	A description of any assumptions or corrections, such as tests of normality and adjustment for multiple comparisons
☐	☒	A full description of the statistical parameters including central tendency (e.g. means) or other basic estimates (e.g. regression coefficient) AND variation (e.g. standard deviation) or associated estimates of uncertainty (e.g. confidence intervals)
☒	☐	For null hypothesis testing, the test statistic (e.g. *F*, *t*, *r*) with confidence intervals, effect sizes, degrees of freedom and *P* value noted<br>*Give P values as exact values whenever suitable.*
☒	☐	For Bayesian analysis, information on the choice of priors and Markov chain Monte Carlo settings
☒	☐	For hierarchical and complex designs, identification of the appropriate level for tests and full reporting of outcomes
☐	☒	Estimates of effect sizes (e.g. Cohen's *d*, Pearson's *r*), indicating how they were calculated

Our web collection on statistics for biologists contains articles on many of the points above.

Software and code

Policy information about availability of computer code

Data collection	MERFISH imaging data was collected using custom Python code to control the microscope. This code is available at https://github.com/ZhuangLab.
Data analysis	The MERFISH data was analyzed using custom Python code. This code is available at https://github.com/ZhuangLab/MERlin.<br>Other packages used in data analyses include: Scanpy (version 1.4); Bioconductor limma (version 3.38); Scrublet (version 0.2).

For manuscripts utilizing custom algorithms or software that are central to the research but not yet described in published literature, software must be made available to editors/reviewers. We strongly encourage code deposition in a community repository (e.g. GitHub). See the Nature Research guidelines for submitting code & software for further information.

Data

Policy information about availability of data

All manuscripts must include a data availability statement. This statement should provide the following information, where applicable:

- Accession codes, unique identifiers, or web links for publicly available datasets
- A list of figures that have associated raw data
- A description of any restrictions on data availability

Data availability statement is included in the manuscript, which states:
The data that support the findings of this study are available from the corresponding author upon reasonable request. Raw and processed MERFISH data can be accessed at the Brain Image Library : https://doi.brainimagelibrary.org/doi/10.35077/g.21.

Field-specific reporting

Please select the one below that is the best fit for your research. If you are not sure, read the appropriate sections before making your selection.

☒ Life sciences ☐ Behavioural & social sciences ☐ Ecological, evolutionary & environmental sciences

For a reference copy of the document with all sections, see nature.com/documents/nr-reporting-summary-flat.pdf

Life sciences study design

All studies must disclose on these points even when the disclosure is negative.

Sample size	Two replicate animals were imaged under each condition. From the two replicate animals imaged for the identification and spatial mapping of cell types, a total of ~300,000 cells were imaged, which generated a sufficient number of single-cell profiles and gave sufficient statistics for the effect sizes of interest. From the two replicate animals imaged for projection target mapping, a total of ~190,000 cells were imaged, which gave sufficient statistics for the effect sizes of interest.
Data exclusions	We did not exclude any data from consideration. All images were included in the primary analysis.
Replication	Reported results were replicated from two animals under each condition.
Randomization	Two male animals were randomly chosen for the identification and spatial mapping of cell types, and two male animals were randomly chosen for the projection pattern study. For each animal, the imaging experiments were definitive and no randomization was necessary for this study.
Blinding	Blinding during collection was not needed because all images were taken under same condition. Blinding during analysis was not necessary because the results were quantitative and did not require subjective judgment. Blinding is not typically used in the field.

Reporting for specific materials, systems and methods

We require information from authors about some types of materials, experimental systems and methods used in many studies. Here, indicate whether each material, system or method listed is relevant to your study. If you are not sure if a list item applies to your research, read the appropriate section before selecting a response.

Materials & experimental systems

n/a	Involved in the study	
☒	☐	Antibodies
☒	☐	Eukaryotic cell lines
☒	☐	Palaeontology
☐	☒	Animals and other organisms
☒	☐	Human research participants
☒	☐	Clinical data

Methods

n/a	Involved in the study	
☒	☐	ChIP-seq
☒	☐	Flow cytometry
☒	☐	MRI-based neuroimaging

Animals and other organisms

Policy information about studies involving animals; ARRIVE guidelines recommended for reporting animal research

Laboratory animals	Adult C57BL/6J male mice aged 57-63 days were used in this study. Mice were maintained on a 12h/12h light/dark cycle, at a temperature of 22 ± 1 °C, a humidity of 30-70%, with ad libitum access to food and water.
Wild animals	The study did not involve wild animals.
Field-collected samples	The study did not involve samples collected from the field.
Ethics oversight	Harvard University Institutional Animal Care and Use Committee;<br>University of South California Institutional Animal Care and Use Committee.

Note that full information on the approval of the study protocol must also be provided in the manuscript.
